# Crystal structure and thermal behaviour of pyridinium styphnate

**DOI:** 10.1107/S2056989014027704

**Published:** 2015-01-03

**Authors:** Selvarasu Muthulakshmi, Doraisamyraja Kalaivani

**Affiliations:** a PG and Research Department of Chemistry, Seethalakshmi Ramaswami College, Tiruchirappalli 620 002, Tamil Nadu, India

**Keywords:** crystal structure, pyridinium, styphnate, TGA/DTA studies, hydrogen bonding

## Abstract

In the crystal structure of the title mol­ecular salt, C_5_H_6_N^+^·C_6_H_2_N_3_O_8_^−^ (systematic name: pyridinium 3-hy­droxy-2,4,6-tri­nitro­phenolate), the pyridin­ium cation and the 3-hy­droxy-2,4,6-tri­nitro­phenolate anion are linked through bifurcated N—H⋯(O,O) hydrogen bonds, forming an *R*_1_^2^(6) ring motif. The nitro group *para* with respect to phenolate ion forms an intra­molecular hydrogen bond with the adjacent phenolic –OH group, which results in an *S*(6) ring motif. The nitro group flanked by the phenolate ion and the phenolic –OH group deviates noticeably from the benzene ring, subtending a dihedral angle of 89.2 (4)°. The other two nitro groups deviate only slightly from the plane of the benzene ring, making dihedral angles of 2.8 (4) and 3.4 (3)°. In the crystal, the 3-hy­droxy-2,4,6-tri­nitro­phenolate anions are linked through O—H⋯O hydrogen bonds, forming chains along [100]. These anionic chains, to which the cations are attached, are linked *via* C—H⋯O hydrogen bonds, forming a three-dimensional structure. Impact friction sensitivity tests and TGA/DTA studies on the title mol­ecular salt imply that it is an insensitive high-energy-density material.

## Abstract 2

In the crystal of the title mol­ecular salt, the pyridinium cation and the 3-hy­droxy-2,4,6-tri­nitro­phenolate anion are linked through bifurcated N—H⋯(O,O) hydrogen bonds forming an 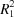
(6) ring motif. Impact, friction sensitivity tests and TGA/DTA studies on this compound imply that it is an insensitive high-energy-density material.

## Chemical context   

A number of crystalline styphnate salts with inorganic metal cations have been reported in recent years (Cui *et al.*, 2008*a* ▸,*b* ▸; Hu *et al.*, 2005 ▸; Liu *et al.*, 2009 ▸; Orbovic & Codoceo, 2008 ▸; Zhang *et al.*, 2011*a* ▸,*b* ▸; Zheng *et al.*, 2006*a* ▸,*b* ▸; Zhu & Xiao, 2009 ▸). In spite of the fact that styphnates with protonated organic amine cations crystallize with difficulty (Vogel, 1978 ▸), they have received attention because of their high thermal stability (Abashev *et al.*, 2001*a* ▸,*b* ▸; Deblitz *et al.*, 2012 ▸; Kalaivani & Malarvizhi, 2010 ▸; Kalaivani *et al.*, 2011 ▸; Kazheva *et al.*, 2002 ▸; Liu *et al.*, 2008 ▸; Refat *et al.*, 2013 ▸; Tang *et al.*, 2012 ▸; Zhang *et al.*, 2012 ▸; Wu *et al.*, 2013*a* ▸,*b* ▸,*c* ▸). Amorphous pyridinium styphnate has found applications in the preparation of chloro­picryl chloride (Feuer & Harban, 1954 ▸). We report herein on the crystal structure of the title mol­ecular salt.
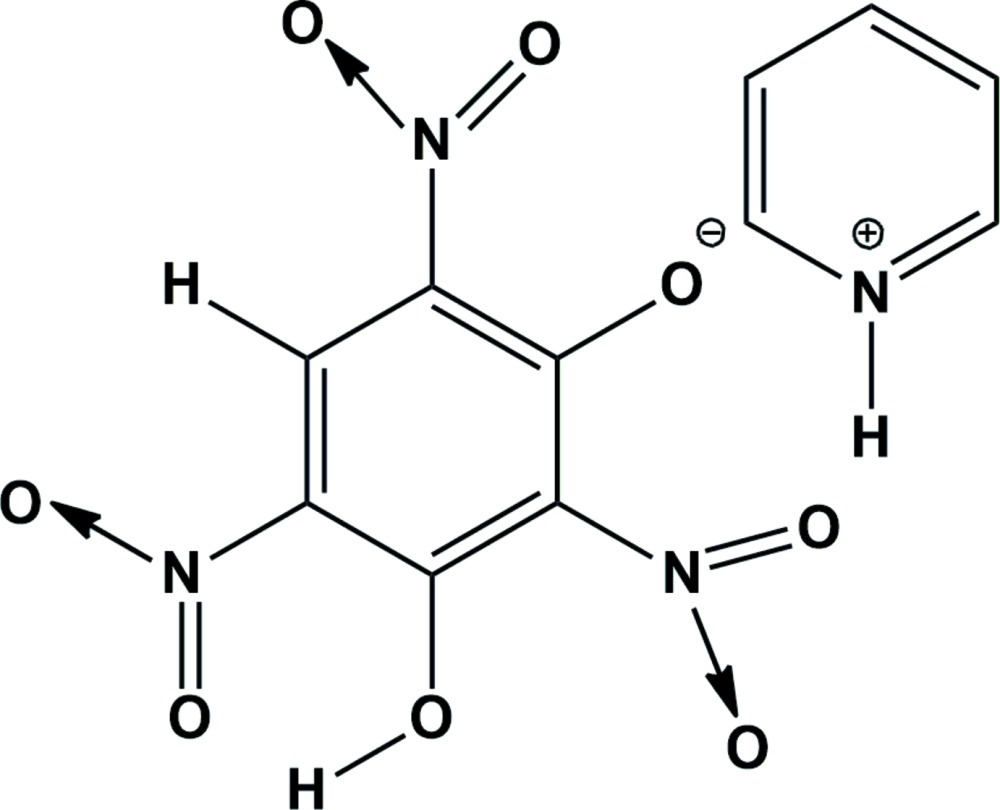


## Structural commentary   

**Figure 1:**
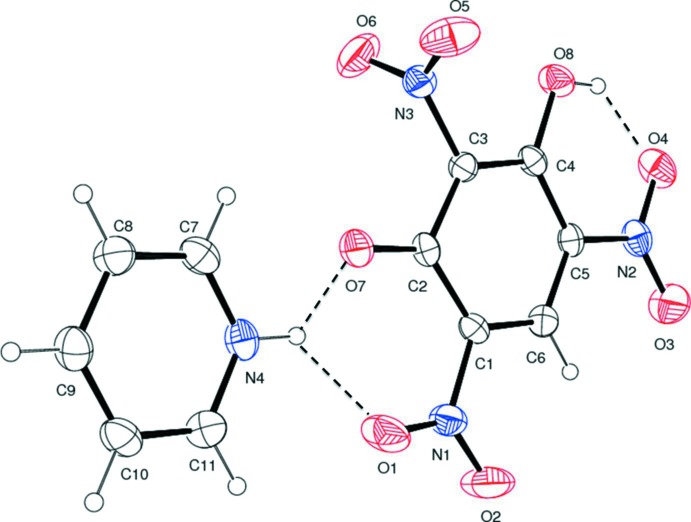
A view of the mol­ecular structure of the title mol­ecular salt, showing the atom labelling. Displacement ellipsoids are drawn at the 30% probability level. Hydrogen bonds are shown as dashed lines (see Table 1 ▸ for details).

The mol­ecular structure of the title mol­ecular salt is depicted in Fig. 1 ▸. The asymmetric unit is comprised of one phenolate anion and a pyridinium cation. The loss of a single proton of the styphnate anion is confirmed by the increase in the bond lengths of the C—C bonds adjacent to the phenolate ion (C1—C2 and C2—C3), which are 1.439 (4) and 1.420 (4) Å, respectively. There is an increase of the value of the bond angles C2—C1—C6 and C2—C3—C4 in the benzene ring to 122.4 (3) and 126.3 (3)°, respectively, and a decrease of the C4—C5—C6 bond angle to 120.5 (2)° compared to the values observed for free styphic acid (Pierce-Butler, 1982 ▸). The nitro group (N3/O5/O6) flanked by the phenolate ion and the phenolic –OH group deviates noticeably from the benzene ring plane, subtending a dihedral angle of 89.2 (4)°. The other two nitro groups, O1/N1/O2 and O3/N2/O4, lie close to the plane of the attached benzene ring, making dihedral angles of 2.8 (4) and 3.4 (3) °, respectively. The nitro group (N2/O3/O4) *para* with respect to the phenolate O atom, O7, forms an intra­molecular hydrogen bond with the adjacent phenolic –OH group (O8—H8), which results in an *S*(6) ring motif (Fig. 1 ▸ and Table 1 ▸).

## Supra­molecular features   

**Figure 2:**
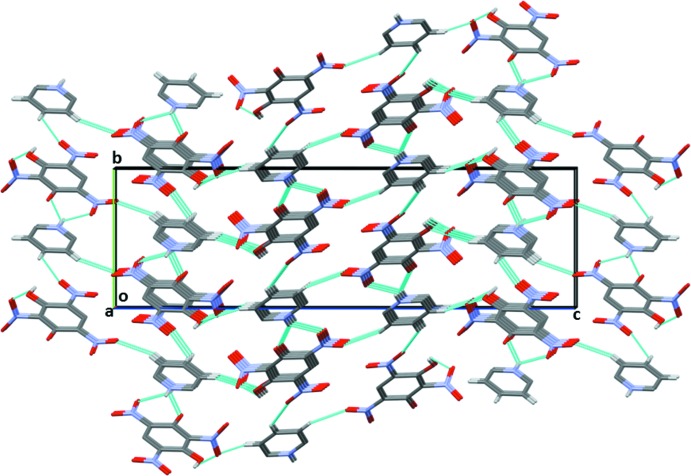
A view along the *a* axis of the crystal packing of the title mol­ecular salt. Hydrogen bonds are shown as dashed lines (see Table 1 ▸ for details).

In the crystal, the cation and anion are linked *via* bifurcated N—H⋯(O,O) hydrogen bonds forming an 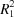
(6) ring motif (Table 1 ▸ and Figs. 1 ▸ and 2 ▸). Inversion-related anions are connected through pairs of C—H⋯O hydrogen bonds, forming dimers enclosing an 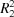
(10) ring motif. The phenolate oxygen, O7, is also bifurcated and forms hydrogen bonds with the protonated nitro­gen atom, N4, of the pyridinium moiety and the C—H H atom adjacent to the protonated nitro­gen atom, forming an 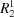
(5) ring motif. The combination of these various N—H⋯O, O—H⋯O and C—H⋯O hydrogen bonds leads to the formation of a three-dimensional structure (Table 1 ▸ and Figs. 2 ▸ and 3 ▸).

## Database survey   

A search of the Cambridge Structural Database (Version 5.35, May 214; Groom & Allen, 2014 ▸) for 3-hy­droxy-2,4,6-tri­nitro­phenolates gave 14 hits. Six concern metal-complex cations, and the remaining eight concern organic cations. Amongst the latter are two compounds, referred to above in §1 for their high thermal stability, viz. 2-meth­oxy­anilinium 3-hy­droxy-2,4,6-tri­nitro­phenolate (Kalaivani *et al.*, 2011 ▸) and morpho­linium 3-hy­droxy-2,4,6-tri­nitro­phenolate (Kalaivani & Malarvizhi, 2010 ▸).

## Thermal behaviour and friction sensitivity   

As styphnic acid derivatives are energetic salts, the thermal behaviour of the title mol­ecular salt has also been examined. The exothermic decomposition has been observed at four different heating rates (5 K/min, 10 K/min, 20 K/min and 40 K/min). The title mol­ecular salt decomposes (70–80%) in two stages. For each stage, the energy of activation was determined employing Kissinger (1957 ▸) [stage I: 27.2 kcal/mol; stage II: 50.5 kcal/mol] and Ozawa (1965 ▸) methods [stage I: 28.5 kcal/mol; stage II: 51.8 kcal/mol]. The title mol­ecular salt was observed to be insensitive towards the impact of a 2 kg mass hammer up to the height limit (160 cm) of the instrument, even at the maximum energy level of 31.392 J (Meyer & Kohler, 1993*a* ▸). The friction sensitivity was determined under defined conditions according to the BAM method (Meyer & Kohler, 1993*b* ▸). The title mol­ecular salt was insensitive at the maximum force of 360 Newton. The title mol­ecular salt is an insensitive high-energy-density material, confirmed through the impact, friction-sensitivity test, and the energy of activation from TGA/DTA curves.

## Synthesis and crystallization   

Styphnic acid (2.45 g, 0.01 mol) dissolved in 25 mL of absolute alcohol was mixed with pyridine (0.79 g, 0.01 mol) and stirred continuously for 6 hrs and then kept aside for 2 h. The yellow-coloured amorphous solid obtained was filtered, washed with 30 ml of dry ether and recrystallized from ethyl­ene glycol. Yellow crystals formed in an ethyl­ene glycol solution after slow evaporation at 298 K over a period of 2 weeks (m.p: 455 K; yield: 80%).

## Refinement   

Crystal data, data collection and structure refinement details are summarized in Table 2 ▸. The NH H atom was located from a difference Fourier map and freely refined. The OH and C-bound H atoms were included in calculated positions and treated as riding atoms: O—H = 0.82, C—H = 0.93 Å, with *U*_iso_(H) = 1.5*U*_eq_(O) for the hydroxyl H atom and = 1.2*U*_eq_(C) for the other H atoms.

## Supplementary Material

Crystal structure: contains datablock(s) global, I. DOI: 10.1107/S2056989014027704/su5046sup1.cif

Structure factors: contains datablock(s) I. DOI: 10.1107/S2056989014027704/su5046Isup2.hkl

Supporting information file. DOI: 10.1107/S2056989014027704/su5046Isup3.cml

CCDC reference: 1006492

Additional supporting information:  crystallographic information; 3D view; checkCIF report

## Figures and Tables

**Figure 3:**
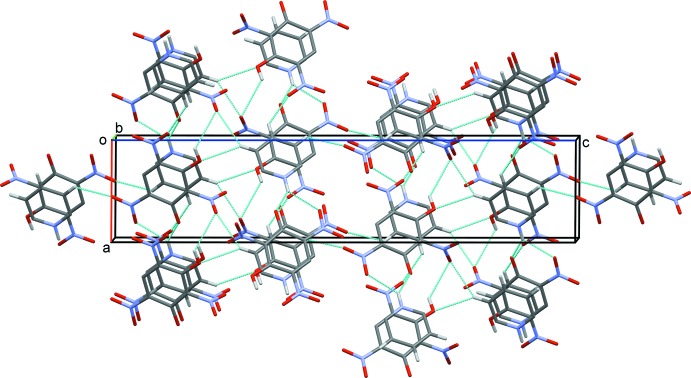
A view along the *b* axis of the crystal packing of the title mol­ecular salt. Hydrogen bonds are shown as dashed lines (see Table 1 ▸ for details).

**Table 1:** Hydrogen-bond geometry (, )

*D*H*A*	*D*H	H*A*	*D* *A*	*D*H*A*
N4H4*A*O1	0.90(2)	2.22(4)	2.946(4)	137(4)
N4H4*A*O7	0.90(2)	1.88(4)	2.625(3)	138(5)
O8H8*A*N2	0.82	2.48	2.905(3)	113
O8H8*A*O4	0.82	1.87	2.563(3)	141
O8H8*A*O6^i^	0.82	2.63	3.110(4)	119
C8H8O6^ii^	0.93	2.58	3.352(5)	141
C8H8O8^iii^	0.93	2.63	3.405(4)	141
C10H10O2^iv^	0.93	2.43	3.139(4)	133

Symmetry codes: (i) 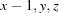; (ii) 
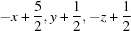
; (iii) 
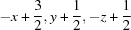
; (iv) 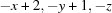.

**Table 2:** Experimental details

Crystal data	
Chemical formula	C_5_H_6_N^+^C_6_H_2_N_3_O_8_
*M*_r_	324.21
Crystal system, space group	Monoclinic, *P*2_1_/*n*
Temperature (K)	296
*a*, *b*, *c* ()	5.9506(2), 8.1608(3), 27.0175(10)
()	90.379(5)
*V* (^3^)	1311.99(8)
*Z*	4
Radiation type	Mo *K*
(mm^1^)	0.14
Crystal size (mm)	0.35 0.35 0.30

Data collection	
Diffractometer	Bruker Kappa APEXII CCD
Absorption correction	Multi-scan (*SADABS*; Bruker, 2004 ▸)
*T*_min_, *T*_max_	0.951, 0.959
No. of measured, independent and observed [*I* > 2(*I*)] reflections	14733, 2296, 1771
*R*_int_	0.047
(sin /)_max_ (^1^)	0.595

Refinement	
*R*[*F* ^2^ > 2(*F* ^2^)], *wR*(*F* ^2^), *S*	0.059, 0.198, 1.14
No. of reflections	2296
No. of parameters	212
No. of restraints	1
H-atom treatment	H atoms treated by a mixture of independent and constrained refinement
_max_, _min_ (e ^3^)	0.32, 0.34

Computer programs: *APEX2*, *SAINT* and *XPREP* (Bruker, 2004 ▸), *SIR92* (Altomare *et al.*, 1993 ▸), *SHELXL97* (Sheldrick, 2008 ▸), *ORTEP-3 for Windows* (Farrugia, 2012 ▸) and *Mercury* (Macrae *et al.*, 2008 ▸).

## supplementary crystallographic information

### Crystal data

**Table:**

C_5_H_6_N^+^·C_6_H_2_N_3_O_8_^−^	*F*(000) = 664
*M**_r_* = 324.21	*D*_x_ = 1.641 Mg m^−^^3^
Monoclinic, *P*2_1_/*n*	Mo *K*α radiation, λ = 0.71073 Å
Hall symbol: -P 2yn	Cell parameters from 5354 reflections
*a* = 5.9506 (2) Å	θ = 2.6–26.1°
*b* = 8.1608 (3) Å	µ = 0.14 mm^−^^1^
*c* = 27.0175 (10) Å	*T* = 296 K
β = 90.379 (5)°	Block, yellow
*V* = 1311.99 (8) Å^3^	0.35 × 0.35 × 0.30 mm
*Z* = 4	

### Data collection

**Table:**

Bruker Kappa APEXII CCD diffractometer	2296 independent reflections
Radiation source: fine-focus sealed tube	1771 reflections with *I* > 2σ(*I*)
Graphite monochromator	*R*_int_ = 0.047
ω and φ scan	θ_max_ = 25.0°, θ_min_ = 2.6°
Absorption correction: multi-scan (*SADABS*; Bruker, 2004)	*h* = −5→7
*T*_min_ = 0.951, *T*_max_ = 0.959	*k* = −9→9
14733 measured reflections	*l* = −32→32

### Refinement

**Table:**

Refinement on *F*^2^	Primary atom site location: structure-invariant direct methods
Least-squares matrix: full	Secondary atom site location: difference Fourier map
*R*[*F*^2^ > 2σ(*F*^2^)] = 0.059	Hydrogen site location: inferred from neighbouring sites
*wR*(*F*^2^) = 0.198	H atoms treated by a mixture of independent and constrained refinement
*S* = 1.14	*w* = 1/[σ^2^(*F*_o_^2^) + (0.1017*P*)^2^ + 0.9268*P*] where *P* = (*F*_o_^2^ + 2*F*_c_^2^)/3
2296 reflections	(Δ/σ)_max_ < 0.001
212 parameters	Δρ_max_ = 0.32 e Å^−^^3^
1 restraint	Δρ_min_ = −0.34 e Å^−^^3^

### Special details

**Table:**

Geometry. All esds (except the esd in the dihedral angle between two l.s. planes) are estimated using the full covariance matrix. The cell esds are taken into account individually in the estimation of esds in distances, angles and torsion angles; correlations between esds in cell parameters are only used when they are defined by crystal symmetry. An approximate (isotropic) treatment of cell esds is used for estimating esds involving l.s. planes.
Refinement. Refinement of F^2^ against ALL reflections. The weighted R-factor wR and goodness of fit S are based on F^2^, conventional R-factors R are based on F, with F set to zero for negative F^2^. The threshold expression of F^2^ > 2sigma(F^2^) is used only for calculating R-factors(gt) etc. and is not relevant to the choice of reflections for refinement. R-factors based on F^2^ are statistically about twice as large as those based on F, and R- factors based on ALL data will be even larger.

### Fractional atomic coordinates and isotropic or equivalent isotropic displacement parameters (Å^2^)

**Table:**

	*x*	*y*	*z*	*U*_iso_*/*U*_eq_	
C1	0.5386 (5)	0.1613 (3)	0.08054 (10)	0.0338 (6)	
C2	0.6344 (5)	0.1587 (3)	0.12957 (10)	0.0335 (6)	
C3	0.5003 (5)	0.0711 (3)	0.16385 (10)	0.0330 (6)	
C4	0.3031 (4)	−0.0084 (3)	0.15364 (10)	0.0331 (6)	
C5	0.2257 (4)	−0.0036 (3)	0.10427 (10)	0.0342 (7)	
C6	0.3424 (5)	0.0820 (4)	0.06890 (10)	0.0372 (7)	
H6	0.2873	0.0862	0.0366	0.045*	
C7	1.2035 (5)	0.4297 (4)	0.17352 (12)	0.0451 (8)	
H7	1.1200	0.3709	0.1964	0.054*	
C8	1.3941 (6)	0.5094 (4)	0.18837 (12)	0.0485 (8)	
H8	1.4407	0.5063	0.2213	0.058*	
C9	1.5148 (6)	0.5936 (4)	0.15407 (12)	0.0476 (8)	
H9	1.6461	0.6474	0.1635	0.057*	
C10	1.4438 (6)	0.5993 (4)	0.10590 (12)	0.0488 (8)	
H10	1.5250	0.6577	0.0825	0.059*	
C11	1.2528 (6)	0.5186 (4)	0.09267 (12)	0.0471 (8)	
H11	1.2027	0.5209	0.0600	0.057*	
N1	0.6481 (5)	0.2501 (3)	0.04118 (9)	0.0477 (7)	
N2	0.0254 (4)	−0.0877 (3)	0.09001 (10)	0.0445 (7)	
N3	0.5834 (4)	0.0636 (3)	0.21455 (9)	0.0436 (7)	
N4	1.1371 (4)	0.4356 (3)	0.12684 (10)	0.0419 (6)	
O1	0.8179 (5)	0.3281 (4)	0.04960 (9)	0.0730 (9)	
O2	0.5667 (5)	0.2440 (5)	0.00022 (10)	0.0953 (12)	
O3	−0.0431 (5)	−0.0774 (4)	0.04783 (9)	0.0732 (8)	
O4	−0.0736 (4)	−0.1705 (3)	0.12168 (9)	0.0589 (7)	
O5	0.5195 (6)	0.1641 (3)	0.24370 (9)	0.0800 (10)	
O6	0.7049 (5)	−0.0493 (5)	0.22574 (10)	0.0913 (11)	
O7	0.8146 (3)	0.2234 (3)	0.14326 (8)	0.0493 (6)	
O8	0.1994 (4)	−0.0845 (3)	0.19071 (8)	0.0478 (6)	
H8A	0.0816	−0.1247	0.1805	0.072*	
H4A	1.006 (6)	0.388 (7)	0.1186 (18)	0.118 (19)*	

### Atomic displacement parameters (Å^2^)

**Table:**

	*U*^11^	*U*^22^	*U*^33^	*U*^12^	*U*^13^	*U*^23^
C1	0.0332 (15)	0.0309 (14)	0.0374 (15)	−0.0026 (12)	0.0065 (12)	−0.0001 (11)
C2	0.0283 (14)	0.0298 (13)	0.0424 (15)	0.0001 (11)	0.0028 (12)	−0.0035 (11)
C3	0.0324 (15)	0.0339 (14)	0.0327 (14)	−0.0014 (12)	−0.0003 (11)	−0.0011 (11)
C4	0.0317 (15)	0.0281 (13)	0.0397 (15)	−0.0009 (11)	0.0066 (12)	−0.0007 (11)
C5	0.0261 (14)	0.0323 (14)	0.0441 (16)	−0.0023 (11)	−0.0006 (12)	−0.0039 (12)
C6	0.0367 (16)	0.0374 (15)	0.0374 (15)	0.0010 (12)	−0.0014 (12)	−0.0043 (12)
C7	0.0468 (19)	0.0385 (16)	0.0503 (18)	−0.0043 (14)	0.0104 (14)	0.0022 (13)
C8	0.053 (2)	0.0475 (18)	0.0455 (17)	−0.0064 (16)	−0.0028 (15)	0.0011 (14)
C9	0.0424 (18)	0.0414 (17)	0.059 (2)	−0.0093 (14)	−0.0024 (15)	−0.0018 (15)
C10	0.0491 (19)	0.0419 (17)	0.055 (2)	−0.0082 (15)	0.0100 (15)	0.0081 (14)
C11	0.053 (2)	0.0450 (17)	0.0432 (17)	−0.0018 (15)	−0.0018 (15)	0.0019 (14)
N1	0.0497 (16)	0.0525 (16)	0.0408 (15)	−0.0080 (13)	0.0037 (12)	0.0065 (12)
N2	0.0343 (14)	0.0446 (14)	0.0544 (16)	−0.0056 (12)	0.0010 (12)	−0.0080 (12)
N3	0.0395 (15)	0.0515 (16)	0.0398 (14)	−0.0069 (12)	0.0005 (11)	0.0036 (12)
N4	0.0349 (14)	0.0362 (13)	0.0547 (16)	−0.0041 (11)	0.0008 (12)	−0.0050 (11)
O1	0.0702 (18)	0.091 (2)	0.0575 (15)	−0.0443 (16)	0.0055 (13)	0.0103 (14)
O2	0.092 (2)	0.147 (3)	0.0465 (15)	−0.054 (2)	−0.0129 (15)	0.0350 (17)
O3	0.0594 (17)	0.102 (2)	0.0584 (16)	−0.0280 (15)	−0.0190 (13)	−0.0023 (15)
O4	0.0472 (14)	0.0603 (15)	0.0692 (16)	−0.0234 (12)	0.0075 (12)	−0.0048 (12)
O5	0.133 (3)	0.0616 (16)	0.0453 (14)	0.0037 (17)	−0.0071 (15)	−0.0118 (13)
O6	0.089 (2)	0.124 (3)	0.0600 (17)	0.052 (2)	−0.0210 (15)	0.0014 (17)
O7	0.0364 (12)	0.0594 (14)	0.0521 (13)	−0.0177 (10)	−0.0026 (10)	0.0031 (10)
O8	0.0440 (13)	0.0533 (13)	0.0464 (12)	−0.0163 (10)	0.0059 (10)	0.0059 (10)

### Geometric parameters (Å, º)

**Table:**

C1—C6	1.369 (4)	C8—H8	0.9300
C1—C2	1.439 (4)	C9—C10	1.366 (4)
C1—N1	1.445 (4)	C9—H9	0.9300
C2—O7	1.249 (3)	C10—C11	1.359 (5)
C2—C3	1.420 (4)	C10—H10	0.9300
C3—C4	1.367 (4)	C11—N4	1.339 (4)
C3—N3	1.454 (4)	C11—H11	0.9300
C4—O8	1.333 (3)	N1—O2	1.206 (4)
C4—C5	1.409 (4)	N1—O1	1.215 (4)
C5—C6	1.376 (4)	N2—O3	1.211 (3)
C5—N2	1.426 (4)	N2—O4	1.242 (3)
C6—H6	0.9300	N3—O5	1.200 (4)
C7—N4	1.320 (4)	N3—O6	1.208 (4)
C7—C8	1.366 (5)	N4—H4A	0.90 (2)
C7—H7	0.9300	O8—H8A	0.8200
C8—C9	1.362 (5)		
			
C6—C1—C2	122.4 (3)	C7—C8—H8	120.6
C6—C1—N1	117.1 (3)	C8—C9—C10	120.3 (3)
C2—C1—N1	120.5 (2)	C8—C9—H9	119.9
O7—C2—C3	120.3 (3)	C10—C9—H9	119.9
O7—C2—C1	126.9 (3)	C11—C10—C9	119.1 (3)
C3—C2—C1	112.8 (2)	C11—C10—H10	120.4
C4—C3—C2	126.3 (3)	C9—C10—H10	120.4
C4—C3—N3	117.1 (2)	N4—C11—C10	119.8 (3)
C2—C3—N3	116.5 (2)	N4—C11—H11	120.1
O8—C4—C3	118.1 (2)	C10—C11—H11	120.1
O8—C4—C5	125.0 (2)	O2—N1—O1	121.5 (3)
C3—C4—C5	116.9 (2)	O2—N1—C1	118.3 (3)
C6—C5—C4	120.5 (2)	O1—N1—C1	120.2 (3)
C6—C5—N2	118.8 (3)	O3—N2—O4	121.9 (3)
C4—C5—N2	120.7 (3)	O3—N2—C5	119.8 (3)
C1—C6—C5	120.9 (3)	O4—N2—C5	118.4 (3)
C1—C6—H6	119.5	O5—N3—O6	123.2 (3)
C5—C6—H6	119.5	O5—N3—C3	118.8 (3)
N4—C7—C8	120.4 (3)	O6—N3—C3	117.8 (3)
N4—C7—H7	119.8	C7—N4—C11	121.7 (3)
C8—C7—H7	119.8	C7—N4—H4A	118 (3)
C9—C8—C7	118.7 (3)	C11—N4—H4A	120 (3)
C9—C8—H8	120.6	C4—O8—H8A	109.5
			
C6—C1—C2—O7	178.0 (3)	N2—C5—C6—C1	−178.3 (3)
N1—C1—C2—O7	−2.3 (4)	N4—C7—C8—C9	−0.6 (5)
C6—C1—C2—C3	−2.0 (4)	C7—C8—C9—C10	0.8 (5)
N1—C1—C2—C3	177.7 (2)	C8—C9—C10—C11	−0.7 (5)
O7—C2—C3—C4	−178.4 (3)	C9—C10—C11—N4	0.3 (5)
C1—C2—C3—C4	1.6 (4)	C6—C1—N1—O2	−3.0 (5)
O7—C2—C3—N3	0.6 (4)	C2—C1—N1—O2	177.3 (3)
C1—C2—C3—N3	−179.4 (2)	C6—C1—N1—O1	177.1 (3)
C2—C3—C4—O8	−179.9 (3)	C2—C1—N1—O1	−2.6 (4)
N3—C3—C4—O8	1.1 (4)	C6—C5—N2—O3	−2.8 (4)
C2—C3—C4—C5	0.3 (4)	C4—C5—N2—O3	177.3 (3)
N3—C3—C4—C5	−178.7 (2)	C6—C5—N2—O4	177.0 (3)
O8—C4—C5—C6	178.3 (3)	C4—C5—N2—O4	−2.9 (4)
C3—C4—C5—C6	−1.9 (4)	C4—C3—N3—O5	−87.2 (4)
O8—C4—C5—N2	−1.8 (4)	C2—C3—N3—O5	93.7 (3)
C3—C4—C5—N2	178.0 (2)	C4—C3—N3—O6	89.1 (4)
C2—C1—C6—C5	0.6 (4)	C2—C3—N3—O6	−90.0 (4)
N1—C1—C6—C5	−179.2 (3)	C8—C7—N4—C11	0.2 (5)
C4—C5—C6—C1	1.5 (4)	C10—C11—N4—C7	0.0 (5)

### Hydrogen-bond geometry (Å, º)

**Table:**

*D*—H···*A*	*D*—H	H···*A*	*D*···*A*	*D*—H···*A*
N4—H4*A*···O1	0.90 (2)	2.22 (4)	2.946 (4)	137 (4)
N4—H4*A*···O7	0.90 (2)	1.88 (4)	2.625 (3)	138 (5)
O8—H8*A*···N2	0.82	2.48	2.905 (3)	113
O8—H8*A*···O4	0.82	1.87	2.563 (3)	141
O8—H8*A*···O6^i^	0.82	2.63	3.110 (4)	119
C8—H8···O6^ii^	0.93	2.58	3.352 (5)	141
C8—H8···O8^iii^	0.93	2.63	3.405 (4)	141
C10—H10···O2^iv^	0.93	2.43	3.139 (4)	133

Symmetry codes: (i) *x*−1, *y*, *z*; (ii) −*x*+5/2, *y*+1/2, −*z*+1/2; (iii) −*x*+3/2, *y*+1/2, −*z*+1/2; (iv) −*x*+2, −*y*+1, −*z*.
